# Perspectives in immunotherapy: meeting report from the “Immunotherapy Bridge”, Napoli, December 5^th^ 2015

**DOI:** 10.1186/s40425-016-0168-3

**Published:** 2016-10-18

**Authors:** Paolo A. Ascierto, Maria Libera Ascierto, Silvia Formenti, Sacha Gnjatic, Hans Hammers, Vera Hirsh, Rolf Kiessling, Ignacio Melero, Rita Nanda, Graham Pawelec, Sandro Pignata, Pedro Romero, Daniel E. Speiser, Bernard A. Fox, Francesco M. Marincola

**Affiliations:** 1 Istituto Nazionale Tumori “Fondazione G. Pascale”, Naples, Italy; 2 Melanoma. Cancer Immunotherapy and Innovative Therapy Unit, Istituto Nazionale Tumori Fondazione “G. Pascale” Via Mariano Semmola, 80131 Napoli, Italy; 3 Kimmel Cancer Center, Johns Hopkins School of Medicine, Baltimore, MD USA; 4 Department of Radiation Oncology, Weill Cornell Medical College, New York, NY USA; 5 Tisch Cancer Institute, Hematology/Oncology, Immunology, Icahn School of Medicine at Mount Sinai, New York, USA; 6 UT Southwestern, Kidney Cancer Program, Dallas, TX USA; 7 McGill University, Royal Victoria Hospital, Montreal, Canada; 8 Department of Oncology/Pathology, Karolinska Institutet, Stockholm, Sweden; 9 Centro de Investigación Médica Aplicada, and Clinica Universidad de Navarra, Pamplona, Navarra Spain; 10 Section of Hematology/Oncology, Department of Medicine, The University of Chicago, Chicago, IL USA; 11 Second Department of Internal Medicine, University of Tuebingen, Tuebingen, Germany; 12 Department of Oncology and Ludwig Cancer Research Center, University of Lausanne, Lausanne, Switzerland; 13 Oregon Health and Science University, Providence Cancer Center, Portland Medical Center, Portland, OR USA; 14 Sidra Medical and Research Centre, Doha, Qatar

## Abstract

Harnessing the immune system and preventing immune escape, the immunotherapy of cancer provides great potential for clinical application, in broad patient populations, achieving both conventional and unconventional clinical responses. After the substantial advances in melanoma, the focus of cancer immunotherapy has expanded to include many other cancers. Targeting immune checkpoints and further mechanisms used by tumors to avoid anticancer immunity, different approaches are under evaluation, including combination therapies.

The first Immunotherapy Bridge meeting focused on various cancer types including melanoma, non-small cell lung cancer, renal cell, breast and ovarian carcinoma, and discussed mechanisms of action of single agents and combination strategies, and the prediction of clinical responses.

## Introduction

The potential of immunotherapy to improve clinical outcome in various solid and haematologic malignancies is based on the ability of the immune system to exhibit both innate (i.e., myeloid and lymphoid cells exerting a rapid effector function) and adaptive responses (driven by T- and B-lymphocytes expressing antigen receptors produced by site-specific somatic recombination and endowed with greater specificity retaining antigen memory) [1]. The approval of sipuleucel-T for the treatment of prostate cancer was the first immuno-therapy to be approved, followed by the approval of ipilimumab (anti-CTLA-4), for the treatment of metastatic melanoma and, later, of the anti PD-1 antibodies nivolumab (in melanoma, non-small cell lung cancer, NSCLC, kidney cancer, head & neck cancer), pembrolizumab (in melanoma, NSCLC and Head & Neck cancer), and talimogene laherparepvec (T-VEC) in melanoma (the first oncolytic viral therapy approved for treatment).

The clinical practice with checkpoint inhibition and pathways activation has highlighted a number of important considerations. Firstly, immunotherapy agents may be associated with patterns of response that differ from those seen with other treatment modalities (i.e., chemotherapy) and so their use may require the development of different response criteria [2]. A response to anti-CTLA-4 or anti-PD-1 may be observed after an initial increase in tumor volume and a reduction in tumor burden may be observed after the appearance of new lesions. These observations suggest that treatment should be continued regardless of an increase in volume of existing lesions or the early appearance of new lesions. Even more remarkable, durable responses was observed even after cessation of treatment, suggesting an ability of immunotherapy to reset the equilibrium between host and tumor [3]. As an example, it is now accepted that ipilimumab therapy in advanced melanoma presents a plateau in the survival curve, beginning at approximately 3 years [4]. Nevertheless, current criteria (e.g., RECIST) might not be appropriate to asses these types of responses. Another important consideration is that immunotherapies can act regardless of patient characteristics and tumor characteristics [5, 6]. Various approaches to identify potential biomarkers for immunotherapies are being evaluated. A promising area of research is the use of various immunotherapies in combination with one another and/or with chemotherapy, targeted agents or radiation.

### Cancer vaccines revisited

Using a vaccination with CpG 7909 (PF 676), a 23-bp oligodeoxynucleotide TLR-9 agonists, and a human Melan-A peptide antigen (analogue, ELAGIGILTV) emulsified in IFA (Incomplete Freund’s adjuvant), rapid and efficient T cell responses were found in all vaccinated metastatic melanoma patients, with a frequency 10-fold higher than those vaccinated without CpG. The- peak response was 7 to 11 days after booster injection. Furthermore, effector memory (and central) T cells expressing perforin, granzyme B, IFNγ and also CD28-effector cells were generated. Finally, the killing of melanoma cells and IFNγ secretion were observed [7, 8]. More recently robust and sustained CD4 and CD8 T cell responses were found to be induced by vaccination with the long synthetic NY-ESO_79–108_ peptide, CpG and IFA (Baumgaertner P: Vaccination of melanoma patients with long NY-ESO-1 peptide and CpG-B elicits robust T-cell responses with multiple specificities and a novel DR-7-restricted epitope, Submitted). Of the three polypeptide-based cancer vaccines in advanced clinical testing of a recombinant fusion nearly full length MAGE-A3 protein, failed the first primary endpoint in both lung cancer and melanoma, while HPV-16 long synthetic peptides (LSPs) obtained 50 % complete responses in premalignant lesions, but no impact on overall survival (OS) was found in cervical carcinoma [9], and, finally, IMA 109 (a mix of 9 short naturally presented peptides) followed a similar pattern as a clinical efficacy signal was detected in a randomized phase II clinical trial in renal cell carcinoma [10], but failed the primary efficacy endpoint in the phase III trial. Hurdles in vaccination with polypeptides include, first of all, relatively weak T cell responses (narrow focused and short lived), then inadequate induction of CD8 T cells by LSPs and recombinant proteins (cross priming), and, finally, local immunosuppression. Polypeptide-based vaccines still need to be optimized, however they already showed that (1) LSP (and proteins) depend on cross-presentation for vaccination, (2) that XCL-1 may be used as vaccine component to target antigen to XDCs and (3) that fusion XCL-1 proteins are interesting candidates to achieve consistently strong CD8 T cell responses and protective immunity. Also, combination with nanoparticle delivery is a promising approach. Regarding the translation into clinic, toll-like receptor (TLR) agonists as polypeptide vaccine adjuvants CpG-ODNs and Poly(I:C) have been shown to dramatically increase the antigen-specific effector T cell (Teff):Treg ratio in the lymphoid organs, skewing immune response in favor of a functional anti-tumor effect. In addition, CpG-ODNs and Poly(I:C) rapidly induce a Th1 polarized cytokine milieu, which fits with a reported mechanism for the adjuvant activity of TLR ligands [11]. On the other hand, HER-2-CD1d or CEA-CD1d fusion proteins efficiently function both as vaccine adjuvants and driving Th1 shifts in the tumor microenvironment, partly through transactivation of NK cells, thus harnessing NKT for vaccination [12].

Unlike viral antigen specific T cell responses, molecularly defined subunit vaccines (antigen and adjuvants) may not efficiently induce long lived memory cells. Yet, robust tumor antigen-specific T cell memory responses are needed to sustain durable tumor responses. A deep understanding of the signals involved in instructing T cell memory differentiation should provide clues for the identification of compounds that, added to subunit vaccines, may modulate the quantity and quality of memory T cells. In this regard, inhibition of mTOR signaling by administration of rapamycin during the expansion phase of a primary anti-viral CD8 T cell response augments the size of the memory T cell pool during the contraction phase [13]. The mTOR signaling branches into two major complexes, mTORC1 and mTORC2. Conditional genetic inactivation of mTORC1 in T cells leads to a profound reduction of the primary CD8 T cell response affecting the generation of both effector and memory T cells. In marked contrast, the conditional deletion of a functional mTORC2 complex leads to decreased differentiation of short lived effector cells and a corresponding increase in the formation of memory effector precursor cells. This translates into enhanced recall responses to rechallenge with antigen [14]. This observation provides the rationale to dissect the signaling events downstream of mTORC2 leading to instruction of memory T cell differentiation. Nuclear accumulation of the transcription factor FOXO1 is one of the key events. Future research should reveal the specific transcriptional reprogramming events controlled by FOXO1 which lead to memory formation. Similar signaling events may operate in human CD8 T cells [15].

### Antigen-specific T cells, MDSCs and survival in melanoma and breast cancer

Monitoring the immune status of cancer patients using peripheral blood assays has several advantages over tumor-infiltrating cells, such as being minimally invasive, repeatable ad libitum, usable to generate functional as well as phenotypic data. The over-riding caveat is that, like any biomarker, it is only relevant if robustly associated with clinical outcome. In a phase II study of a 10-peptide GM-CSF-adjuvant vaccine in renal cell cancer (RCC) with or without the addition of single low-dose cyclophosphamide, Walter and colleagues showed that a T cell response to 3 or more vaccine peptides was indicative of 100 % survival [10]. Observed reductions of peripheral Tregs with a single dose of cyclophosphamide suggests that suppressive cells might be involved in the response to cancer antigens in these patients. Also, the relative abundance of cells with the phenotype CD14 + HLA-DR^low/negative^ influenced survival in the RCC [10]. Stage IV melanoma patients, on the other hand, possessed higher frequencies of circulating CD4+ Tregs relative to healthy controls, but there was no correlation with survival [16]. In the same study, it was found that the frequencies of CD14 + CD11b + HLA-DR−/low monocytic myeloid-derived suppressor cells (MDSCs) were higher in patients compared to controls and that their levels did correlate negatively with survival. The potential for enhancing the strength of this correlation of MDSC with survival by defining this cell type more accurately was investigated in a pilot study using 45-channel mass spectrometry flow cytometry (CyTOF) on cells preselected for being CD3-, CD19-, CD11b+, CD14+ and CD45+. Comprehensive immune signatures including MDSCs were found in patients relative to controls, which correlated more closely with survival (Wistuba-Hamprecht et al. under revision).

Importantly, the in vitro reactivity of peripheral T cells to tumor-associated antigens (TAA) such as NY-ESO-1 in patients with melanoma with distant metastases is positively associated with a survival benefit [17]. Indicating the complexity of peripheral immune signatures and their correlations with survival, CD8+ T cell reactivity to Melan-A was also associated with survival, but only in the absence of a response of CD4+ T cells producing IL 4 or IL 17 on stimulation with this TAA. Consequently, the presence of CD4+ Melan-A-specific T-cells was in general associated with poorer survival [18]. Similarly, in breast cancer, survival was associated with a CD8+ T cell response to Her-2 peptides and a negative role of both MDSCs and Tregs was demonstrated in the prognosis of these elderly breast cancer patients. The mechanism might be through dampening favorable CD8+ T cell immune responses to tumor-associated antigens [19].

In summary, functional responses of patient peripheral blood to tumor antigens measured in vitro, as well as surface marker phenotyping ex vivo, can predict survival duration. Specificity of the response, nature of the responding T cells and pro- or anti-inflammatory type of response, as well as proportions and types of MDSCs, are all important factors for patient survival in both melanoma and breast cancer. Thus, immune monitoring of peripheral blood identifies predictive biomarkers for patient survival and may provide insights into the mechanisms involved in immune-mediated anti-cancer effects.

### Counteracting immune suppression by modulation of checkpoint blockade and inhibitors of CSF-R1

One of the methods used to improve adoptive T cell therapy is the re-stimulation of the injected TIL (Tumor-infiltrating lymphocyte) cells with tumor vaccine expressing same antigen as recognized by TILs to improve life-span. In a phase I clinical trial Poschke et al. [20] combined a dendritic cell (DC) vaccination and TIL infusion in malignant melanoma. The treatment with the two cellular products was found feasible and safe for the eight patients with stage IV melanoma enrolled in the trial. Although the small study size precluded analysis of clinical responses, one patient showed a complete remission and two had stable disease. The analysis of T-cell receptor repertoire in TIL by next-generation sequencing revealed presence of highly dominant clones in most infusion products which could, in the majority of cases, be detected in the circulation by qPCR after T-cell transfer. Another trial combining DC vaccination and adoptive T cell transfer in a two-step approach in patients preconditioned with non-myeloablative chemotherapy is ongoing (EUdrac-CT #2012-000450-63).

Also, a combination of IFN-γ and multiple Toll-like receptor agonists proved superior in activating DCs to tumor-specific activation, thus permitting an improvement of the quality of the DC vaccine. Even the possibility of combining adoptive T cell transfer with silencing checkpoint molecules was explored. Antibodies represent one possibility, but another intriguing option is a new technology by which the checkpoint molecules could be directly silenced in the injected T cells. Hydrophobically modified “self-deliverable” small RNA (sdRNA) can effectively enter cells and results showed that PD-1 silencing in healthy T cells by using sdRNA leads to significant decrease in surface and as well intracellular levels of PD-1 expression in TILs. Activated PD-1 silenced healthy T cells and TILs produced more IFN-γ upon tumor specific stimulation. In addition, PD-1 silenced TILs proved enhanced capacity to kill autologous tumor cells and, finally, the percentage of CD45RA- CCR7- effector memory CD8+ and CD4+ TILs increased after PD-1 silencing. MDSCs are being evaluated as a new target for cancer immunotherapy and one of the unanswered questions is whether checkpoint blockade mediate some of their effects by targeting immune suppressive cells (Tregs, MDSCs, macrophages) or their products. Anti-CTLA4 treatment results in a strong reduction of immuno-suppressive cells (GrMDSC, Tregs) and agents (Arginase, iNOS) in the majority of treated patients [21].

Neuroblastoma is the most common extracranial solid cancer type in childhood, and high-risk patients have poor prognosis. Similar to malignant melanoma, neuroblastoma-driven inflammation contributes to the induction of suppressive myeloid cells that hamper efficient antitumor immune responses. Infiltrating CSF-1R^+^ myeloid cells predicted poor clinical outcome in patients with neuroblastoma (R2: microarray analysis and visualization platform [22]). In vitro, neuroblastoma-derived factors interfered with early development of myeloid cells and enabled suppressive functions on human monocytes through M-CSF/CSF-1R interaction [23]. In a transgenic mouse model (TH-MYCN) resembling high-risk human neuroblastoma, antagonizing CSF-1R with a selective inhibitor (BLZ945, Novartis) was found to modulate the induction of human and murine suppressive myeloid cells and to efficiently limit tumor progression. Checkpoint inhibitors as monotherapy was found to be insufficient in controlling tumor growth, while combining BLZ945 with PD-1/PD-L1 blocking antibodies resulted in superior tumor control.

### Response and resistance to PD-1 pathway blockade: clues from the tumor microenvironment

Monoclonal antibodies blocking anti-PD-1 immune checkpoint showed an unprecedented spectrum of activity versus different cancer types providing a “common denominator” for cancer therapy [24]. On this note, the US food and drug administration (FDA) already approved their usage for the treatment of patients with metastatic melanoma, advanced NSCLC, RCC and Hodgkin’s lymphoma. Great attention is now focused on identifying biomarkers able to predict response or resistance to anti-PD-1 treatment [25]. It has been previously identified that PD-L1 expression on the tumor cell surface is one factor associated with the clinical activity of anti–PD-1 [26]. Notably, a significant number of patients with PD-L1+ expression still do not respond to PD-1 pathway blockade, suggesting that additional intratumoral factors may influence treatment outcomes.

In melanoma, by comparing PD-L1+ vs. PD-L1(−) tumors, it has been shown an overexpression of genes involved in CD8^+^ T-cell activation (e.g., *CD8A*, *IFNG*) and immunosuppression pathways (e.g., PD-1, *LAG3* and *IL10*) in PD-L1+ tumors, thus suggesting the coordinate expression of multiple immunological factors mediating therapy response to anti PD-1 blockade [27]. However, a deep molecular analysis performed on formalin-fixed, paraffin-embedded (FFPE) pretreatment tumor biopsies expressing PD-L1 (PD-L1+) and derived from patients with RCC showed that none of the immune molecules previously found to be significantly overexpressed in PD-L1+ melanomas was associated with anti-PD-1 treatment response. On the contrary, additional immunologic factors and novel metabolic molecules were found to be associated with treatment resistance in RCC. Particularly, genes involved in metabolic and solute transport functions, also found in kidney cancer cell lines, were found associated with treatment failure of patients with PD-L1+ RCC. Conversely tumors from responding patients overexpressed immune markers such as *BACH2*, a regulator of CD4+ T cell differentiation, and CCL3 involved in leukocyte migration [28]. Thus, the intratumoral balance between metabolic and immunologic gene expression might determine the effective response to anti-PD-1 blockade in RCC. This is not surprising if we consider that RCC has been characterized as a metabolic disease, with the signature up-regulation of factors adapting to hypoxia and functioning to meet the bioenergetic demands of cellular proliferation [29]. These evidences suggested that the general approach used to identifying markers predicting clinical response to anti-PD-1-targeted therapies, which has mostly focused on modulatory receptors and ligands (e.g., PD-1, PD-L2, LAG-3) and T cell infiltrates, must be revaluated. Indeed a deeper level of investigation might be warranted for individual tumor types to which these new therapies are being applied.

### Understanding and combining CD137-targeted immunotherapy

Hepatocellular carcinoma (HCC) is typically an inflammation-associated cancer that can be immunogenic [30]. Association of hepatitis C and hepatitis B infection with upregulation of PD-1 has been reported [31, 32], but it is associated with poor outcomes [33]. Immune checkpoint inhibition (anti-CTLA-4) has shown encouraging activity in an early clinical trial in HCC [34] and also PD-1 blockade with nivolumab may boost host immunity against HCC and improve clinical outcomes. Preliminary data from a phase I/II study on safety and antitumor activity of nivolumab show durable though partial response and an OS rate at 6 months of 72 % [35].

CD137 is a surface glycoprotein involved in T cell co-stimulation, with functions in T cell proliferation, inhibition of apoptosis, cytotoxic activity enhancement and cytokine production. Treatment with anti-CD137 agonist monoclonal antibodies can overcome tumor antigen tolerance [36]. On the other hand, mouse experimentation provided both a rational and a recipe for immunotherapy combinations: (1) procedures aimed at enhancing the number of primed nonanergic T cells in the organism by means of vaccines, adoptive transfer, or procedures to enhance the immunogenicity of a tumor lesion, and (2) strategies to de-repress or stimulate artificially the ongoing immune response, for the most part considering immunostimulatory monoclonal antibodies [37]. Based on this, co-infusion of both agonist antibodies and T cells has been shown to attain marked synergistic effects that result from more focused and intense cytolytic activity visualized under in vivo microscopy and from more efficient entrance of T cells into the tumor through the vasculature. These several levels of dynamic interaction between adoptive T cell therapy and CD137 offer much opportunity to raise the efficacy of current cancer immunotherapies [38]. In particular, anti-CD137 infusion may enhance adoptive immunotherapy by CTL by a dual mechanism, enhancing endogenous effector functions as well as focusing and prolonging the efficacy of therapeutically transferred lymphocytes [39]. New studies, including cross-priming of CD137 with DC and combination of immunostimulatory monoclonal antibodies anti-hCD137 (urelumab) with anti-hPD-1 (nivolumab) are presenting promising results.

### Combination immunotherapy with autologous stem cell transplantation, protein immunization, and PBMC reinfusion in myeloma patients

Multiple myeloma is rich in tumor antigens of the cancer-testis (CT) family conferring spontaneous immunogenicity, with CT7 (MAGE-C1) and MAGE-A3/6 already known to correlate with plasma-cell proliferation [40] and with NY-ESO-1 eliciting spontaneous humoral and T-cell immunity [41]. Antibody responses to CT antigens were found after treatment with allogeneic stem cell transplant in multiple myeloma patients, which might help to achieve long-lasting remissions [42]. Autologous Stem Cell Transplant (ASCT) is a standard of care for some multiple myeloma patients but not curative. ASCT might be used as a platform for adoptive immunotherapy, considering that myeloablative therapy may provide immunologic benefits, such as tumor antigen release for cross-presentation and priming, lymphopenia-induced proliferation of T cells, and elimination of suppressor populations (Tregs, MDSCs). Nevertheless, ASCT also results in durable immunosuppression, as evidenced by frequent CMV reactivation. Combining vaccination and ASCT was therefore investigated for CT antigen MAGE-A3, taking into account potential benefits and risks of MAGE-A3 targeting in adoptive transfer, reported to lead to clinical tumor regression in 5 out of 9 metastatic cancer patients (7 with metastatic melanoma, 1 with synovial sarcoma, 1 with esophageal cancer) but also neurological toxicity associated with lymphocytic brain infiltration, probably due to cross-reactivity with an epitope from MAGE-A12 expressed in human brain [43]. A pilot study of MAGE-A3 antigen-specific cancer immunotherapeutic (recMAGE-A3 protein + AS15) as consolidation for multiple myeloma patients undergoing ASCT has recently completed accrual, and was safe and well tolerated. Using mass cytometry for phenotypic analysis of immune reconstitution, preliminary results show an inversion of CD4 / CD8 ratio and a B cell dip and rebound after ASCT. Nevertheless, antigen-specific assays (ELISA, ELISPOT, Intracellular cytokine staining) for MAGE-A3 showed induction of a strong antibody and Th1-biased CD4 T cell response in all patients. In conclusion, a combined approach of vaccination with ASCT is feasible and overcomes typical immunosuppression in multiple myeloma patients. Absence of MAGE-A expression observed in some patients upon disease relapse suggests potential immune selective pressure, and calls for combination strategies to limit antigen escape.

### Anti-cancer immunity despite T cell “exhaustion”

The three main stumbling blocks for anti-cancer T cells are (1) low T cell numbers and low TCR affinity (with ACT and vaccination as possible solutions), (2) inefficient priming/boosting (with checkpoint blockade or vaccination as possible solutions), and (3) T cell suppression in the TME (with checkpoint blockade, radiation, or intratumoral injection of “adjuvants” as possible solutions) [44].

Tumor antigen specific T cells in the TME only produce low levels of cytokines, thus their effector functions are relatively weak. For the first time in any species, the Speiser group analyzed T cells from a TME by gene expression array [45], demonstrating that the molecular profile significantly resembled the one of T cell “exhaustion” that was first defined in chronic mouse and then human infections [46]. During chronic infection, T cells progressively lose their functional properties. T cell exhaustion is also accompanied by a progressive increase in the amount and diversity of inhibitory receptors expressed. Altered inflammation and changes in immunoregulatory cytokines can have important roles. Ultimately, if the severity and/or duration of the infection is high and/or prolonged, virus-specific T cells can be completely eliminated, leading to loss of protective T cell responses. The severity of T cell exhaustion correlates with inhibitory receptor expression, viral (and antigen) load, loss of CD4+ T cell help, and infection duration. Similar observations were made for metastatic melanoma, the disease studied by the group. They showed this by molecular profiling of CD8+ T cells not only in humans, but also in an autochthonous mouse melanoma model, finding several corresponding molecular hits [45, 47, 48]. They also demonstrated a remarkable coexistence of fully functional effector cells in circulation and exhausted cells in the tumor environment. An important hit, i.e., a gene that was significantly overexpressed in TIL was Maf, an AP-1 family member. Subsequent experiments demonstrated that Maf is a first in-class driver of exhaustion [47, 48], as Maf overexpression reproduces and enhances a large number of molecular and functional features of exhausted cells, and deletion of Maf reverses exhaustion as T cells become strongly functional and eliminate cancers.

Another aspect studied by the group is that exhausted cells still have some functional competence, indicating that exhaustion is not an optimal term, T cell “dysfunction” might be better, or “long term effector T cell population”, because these cells are heterogeneous and likely contain short and (very) long lived cell subsets [49]. In fact, absence of “exhausted” cells renders diseases worse, both in chronic infections and in various cancers. Therefore, the group proposes that exhausted cells are specialized with respect to differentiation and activation, adapted to the disease in which these T cells stably adjust their effector capacity to the conditions of chronic infection or cancer. This phenotype is optimized to cause minimal tissue damage while still mediating a critical level of pathogen control. This concept provides a rationale for emerging therapies that enhance T cell activity in chronic infection and cancer e.g., by blocking inhibitory receptors [49]. Clearly, many dysfunctional cells can regain function within short time, as revealed by strong cytotoxicity by those T cells [50].

The group also studied T cell responses from melanoma patients treated with the anti-CTLA-4 antibody Ipilimumab. Long-term observations suggest that responder patients progressively had less Tregs in blood, and particularly also in tumors. Furthermore, responders had higher levels of non-classical CD16+ monocytes compared with non responder patients. As CD16 binds Ipilimumab, experiments were performed to determine whether these CD16+ monocytes could mediate antibody-dependent cell- mediated cytotoxicity (ADCC). Indeed, this was the case, leading to specific killing of Tregs due to their constitutive CTLA-4 expression at high levels [51]. Another paper, a collaborative work performed in the lab of Pia Kvistborg, Ton Schumacher et al., reports the analysis of melanoma patients’ PBMC with an HLA-A2 tetramer panel comprising 145 tumor antigens. The study shows that anti–CTLA-4 treatment induced a significant increase in the number of detectable CD8 T cell specificities. Thus, the therapy induced considerable broadening of the immune response, demonstrating that Ipilimumab may not primarily act on effector cells but rather on other immune cells, likely Tregs and DCs that can mobilize many novel T cell populations that help fighting cancer [52]. Another paper describes that melanoma patients treated with ipilimumab experienced a significant increase in the number of detectable CD8 T cell specificities. Thus, the therapy induced considerable broadening of the immune response, demonstrating that ipilimumab may not primarily act on already activated effector T cells but rather on other immune cells, likely Tregs and DCs that can activate many novel T cell populations that help fighting cancer [52]. This study was a collaborative work performed in the lab of Pia Kvistborg, Ton Schumacher et al., who developed and applied an HLA-A2 tetramer panel detecting CD8 T cells with 145 different antigen specificities.

### Predictor of response to radiation and immunotherapy

An in vitro model recently showed that radiotherapy (RT) produces both a radiation dose-dependent induction and chemotherapeutic enhancement of immunogenic cell death (ICD) as well as induction of ICD by the combination of radiation and certain chemotherapy agents [53]. These findings provide preliminary evidence that ICD stimulated by either high-dose radiotherapy alone, or concurrent chemo-radiation regimens, may contribute to the establishment of a peritumoral proimmunogenic milieu [53]. In combination with different forms of immunotherapy, RT stimulates the release of antigens and also the signals in the tumor microenvironment that allow T cells to permeate the surviving tumor, and contribute to cell killing so that the tumor is converted into an in situ vaccine. Since 2004, the combination of DC growth factors in combination with RT were found to induce abscopal effects in a syngeneic mouse model [54]. These findings were translated to the clinic, where a combination of radiotherapy with granulocyte-macrophage colony-stimulating factor produced objective abscopal responses in 11 out of 41 patients with metastatic solid tumors refractory to treatment [55]. The results of this clinical trial also highlighted the importance of role of host’s “immune integrity” in the process, with more patients responding among those with a neutrophil to lymphocyte ratio <4. A similar translation of preclinical to clinical results was demonstrated with a combination of immune checkpoint blockade and radiotherapy. In the 4 T1 syngeneic mouse model of metastatic mammary carcinoma, it was shown that RT to a peripheral tumor and CTLA-4 blockade achieved both reduction of lung metastases and improved survival compared either modality alone [56]. Consistently, in a patient with chemotherapy-refractory metastatic adenocarcinoma of the lung, an abscopal response was seen 2 ½ months after the start of treatment with ipilimumab and fractionated RT, supporting the belief that a combination of RT and immunotherapy might prove to be a useful strategy to improve the outcomes of some cancer patients with metastases that are historically known to have dismal prognoses [57].

RT alone is seldom capable of inducing rejection of aggressive poorly immunogenic tumors. RT ability to induce tumor rejection is dependent on the balance of positive and negative signals that pre-exist or are induced by RT itself. Barriers to the potential of radiation to convert a tumor into an in situ vaccine might be associated with classical immunosuppressive microenvironment of established tumors or be RT-induced immunosuppressive effects. One of the immunosuppressive signals generated by RT is the activation transforming growth factor (TGF) β [58]. Because TGFβ regulates many processes that can affect tumor growth, including angiogenesis, a comprehensive assessment of the major changes by gene expression analysis was performed. The top 500 genes selectively upregulated in RT + TGF beta blockade treatment group were identified using the Pavlidis Template Matching Method, defining a majority of genes involved in the immune response. The concept was investigated in a trial of RT with fresolimumab in metastatic breast cancer, that failed to show responses with the combination. The role of adaptive immune resistance was in limiting tumor response to radiotherapy and TGFβ blockade was investigated in a mouse model: increased PDL-1 and PDL-2 expression on tumor and myeloid cells was demonstrated, compared to controls. PD-1 blockade extended survival and delayed tumor recurrence in mice treated with radiation and TGFβ blockade [59], suggesting that PD-1 blockade may be required to unleash the effect of combining TGFβ blockade and RT. In summary, results of RT and immunotherapy combinations from preclinical experiments in syngeneic models of different murine tumors were consistently confirmed in the clinic. RT remains a powerful adjuvant to multiple immunotherapy strategies.

### Checkpoint inhibitors in metastatic non-small cell lung cancer

With limited current options for the treatment of advanced NSCLC and phase III studies of vaccines that to date have failed to meet their primary endpoint, great attention is being paid to checkpoint inhibitors. A double-blind, phase 2 randomized controlled trial in treatment-naïve patients with advanced NSCLC, proved that phased ipilimumab plus chemotherapy improved both irPFS (immune-related PFS) and PFS. Overall rates of grade 3 and 4 immune-related adverse events were 15 % (control 6 %) [60]. Nivolumab monotherapy in a phase II single arm trial found meaningful activity (15 % ORR) and a manageable safety profile (17.1 % grade 3/4 adverse events) in previously treated patients with advanced, refractory, squamous NSCLC [61]. Nivolumab was tested versus chemotherapy in a multicenter, open-label, phase III, randomized trial in previously-treated patients with advanced or metastatic squamous NSCLC, and overall survival (mOS 9.2 vs 6.0), response rate, and progression-free survival were significantly better with nivolumab, regardless of PD-L1 expression level [62]. The corresponding trial in metastatic non-squamous NSCLC (multicenter, open-label, phase III, randomized trial in previously-treated patients) was stopped earlier due to efficacy and nivolumab was associated with even greater efficacy (e.g., mOS 12.2 vs 9.4) than docetaxel across all end points in subgroups defined according to prespecified levels of tumor-membrane expression of the PD-1 ligand. Treatment-related adverse events of grade 3 or 4 were reported in 10 % of patients in the nivolumab group, as compared with 54 % of those in the docetaxel group [63].

The phase I study of pembrolizumab in monotherapy in patients with advanced NSCLC, it had an acceptable side-effect profile and showed antitumor activity in patients (median duration of response exceeds 1 year). Also, PD-L1 expression in at least 50 % of tumor cells correlated with improved efficacy of pembrolizumab [64]. A multicenter, open-label, phase II/III, randomized trial in previously-treated patients with PD-L1-positive NSCLC, demonstrated that pembrolizumab prolongs overall survival (mOS 10.4 with pembrolizumab 2 mg/kg, 12.7 with pembrolizumab 10 mg/kg, 8.5 with docetaxel) and has a favorable benefit-to-risk profile in this selected population [65]. In the multicenter, open-label, phase 3, randomized trial in treatment-naïve patients with PD-L1+ advanced or metastatic NSCLC, pembrolizumab showed a manageable safety profile and promising antitumor activity [66]. Preliminary data from a phase II trial of pembrolizumab in untreated brain metastases in NSCLC showed antitumoral activity (33 % brain metastasis response rate) and good tolerability [67]. In a multicenter, open-label, phase II, randomized trial in previously-treated patients with NSCLC, a pattern of improved survival that correlates with PD-L1 expression and a good safety profile were reported.

### PD1/PDL1 Inhibition in metastatic renal cell carcinoma

The phase III study of nivolumab versus everolimus demonstrated an overall survival benefit for patients with advanced RCC treated with nivolumab, (mOS 25.0 vs 19.6) and was generally well tolerated with a low rate of grade 3/4 events. Prespecified subgroup analyses did not identify a population which did not benefit from nivolumab in terms of overall survival (including patients with PD-L1 negative tumors). On the other hand, a clinically meaningful and statistically significant improvement in quality of life was seen with nivolumab versus everolimus for the duration of the study [68]. The role of treatment beyond progression which was described initially in a phase II dose ranging study in RCC, is still being explored at this time since some patients experience pseudoprogression on PD1 inhibition [69]. The dual immune checkpoint blockade with nivolumab /ipilimumab therapy is showing an encouraging activity with unprecedented ORR of about 40 % [70]. The combination of nivolumab with the vascular endothelial growth factor (VEGF) pathway inhibitors sunitinib or pazopanib in mRCC has shown promising activity as well, but was associated with significant liver toxicity [71]. The association of immunotherapy with more selective VEGF pathway inhibitors was investigated in a phase Ib study with MPDL3280A and bevacizumab with good safety and efficacy outcomes [72]. Phase II and III trials combining immunotherapy and VEGF pathway inhibitors are currently ongoing and will most likely transform first line therapy in the near future. However, a number of questions (e.g., regarding who benefits the most, biomarkers availability, dosing schedule/cost saving opportunities) still need to be answered.

### Immune checkpoint inhibition for breast cancer

Breast cancer is the most common malignancy diagnosed in women worldwide. Approximately 15 % of breast cancers are triple-negative. As triple-negative breast cancers (TNBC) lack expression of ER, PR, and HER2, they do not benefit from available targeted therapies. TNBC is associated with a younger age at diagnosis, an advanced stage at diagnosis, and a worse clinical outcome. The rationale for investigating immunotherapy for TNBC comes from a number of observations made over the past several years. For one, we know that ER negative tumors have a higher density of tumor infiltrating lymphocytes (TILs) than ER positive tumors [73], which can in turn facilitate and immune response. Mittendorf and colleagues found that TNBCs have increased PD-L1 expression, which can suppress T cell function [74]. Gene expression profiling has revealed at least six distinct subtypes of TNBC, including an immunomodulatory subtype which is characterized by elevated expression of genes involved in T cell function [75]. And finally, the genomic instability and high mutation rates in TNBCs can produce neoantigens that induce an immune response [76]. Based on these observations, immune checkpoint inhibition was studied in TNBC. The phase Ib multi-cohort KEYNOTE-12 study enrolled patients with PD-L1 positive TNBC. The 32 patients enrolled were treated with single-agent pembrolizumab at 10 mg/kg every 2 weeks, which demonstrated an acceptable safety profile (events of a potentially immune-mediated nature, regardless of attribution, included pruritus, hepatitis, and hypothyroidism), and was associated with prolonged responses in 18.5 % of patients enrolled in the study [77]. Preliminary data from a phase Ia study of the PD-L1 inhibitor atezolizumab demonstrated a similar safety and tolerability profile, as well as early signs of durable clinical activity with a RR of 19 % [78]. PD-1 or PD-L1 blockade in TNBC is associated with a response rate approaching 20 %, and while somewhat lower than the RR in melanoma (30-40 % range), is comparable to monotherapy response rates in other solid tumors. The responses are durable and immune checkpoint inhibitors are safe and tolerable, with the majority of side effects being mild and easily managed. Future work is aimed at building on these promising monotherapy responses with the combination of different checkpoint inhibitors or with radio/chemo-therapy.

### The role of immunotherapy in ovarian cancer

Despite advances in combinatorial chemotherapy regimens, current therapeutic options for ovarian cancer (OC) patients are inadequate and the majority of patients will relapse and die of disease, with a 5-year survival of approximately 30 %. The strong presence of TILs is strongly, positively associated with improved survival, the activity of TILs (CD3+ and CD8+ cells) as antitumor effectors and the identification of tumor-associated antigens (TAAs) that up-regulate in tumor tissue and ascites of OC patients (Her2/neu, Folate Receptor α, p53, CA125, members of the cancer-testis antigen family like MAGE –A4 and NY-ESO-1), all account for the evidence of OC immunogenicity.

The immunohistochemical analysis of frozen specimens from advanced-stage ovarian carcinomas showed significant differences in the distributions of progression-free survival and overall survival according to the presence or absence of intratumoral T cells. Patients whose tumors contained T cells had a median duration of progression-free survival of 22.4 months and a median duration of overall survival of 50.3 months, as compared with 5.8 and 18.0 months, respectively, among patients whose tumors did not contain T cells [79]. Later, intraepithelial CD8+ tumor-infiltrating lymphocytes and a high CD8+/regulatory T cell ratio were found to be associated with favorable prognosis in OC (mOS 55 vs 26 months) [80]. When taking into account selected published studies that evaluated the association between TIL and patient survival, the prognostic value of TILs persisted among populations with diverse histologic tumor subtypes, regardless of the stage or grade of disease [81]. The Cancer Genome Atlas findings on OC showed that there are few recurrent mutated genes, with four transcript subtypes and the presence of extensive defects homologous recombination with mutual exclusivity [82]. Considering that BRCA 1/2 mutated OC are known to be associated with improved survival and that the presence of TILs (CD3+ and CD8+) and high CD8+/CD4+ ratio are associated with favorable OS in OC, a pilot study explored the differences in the presence of TILs and PD-1 and PD-L1 expression between BRCA mutated and non mutated. The results showed a significantly higher CD8+/CD4+ ratio in BRCA1/2-mutated tumors and peritumoral CD3+ T-cells significantly increased in BRCA1/2-mutated compared to non-BRCA1/2-mutated ovarian cancers. An elevated number of CD3+ and CD8+ in BRCA1/2-mutated OC may provide an additional explanation for the improved clinical outcomes associated with these mutations. In patients with platinum-resistant OC receiving nivolumab, a phase II trial on a very limited number of patients found that nivolumab at 1 mg/kg was well tolerated and has encouraging clinical efficacy [83].

In a phase Ib, open-label expansion trial in patients with previously treated, recurrent or refractory ovarian cancer, avelumab demonstrated an acceptable safety profile and to be clinically active [84]. Activity of avelumab was higher in patients with platinum sensitive recurrence compared to the resistant and in patients with less line of previous chemotherapy. The interim results from a phase Ib study with pembrolizumab in patients with PD-L1 positive advanced OC, has also demonstrated antitumor activity in heavily pretreated patients and a manageable safety and toxicity profile [85]. The first phase 2 and 3 trials are ready to start also in ovarian cancer addressing the role of immunotherapy in all treatment settings of the disease. In the next future it will be also important to identify the dominant immunosuppressive pathways in the different OC subtypes that will allow patients to be optimally matched with therapies. Also, combination strategies should be explored with standard and emerging therapies.

## Conclusions

Based on the characteristics of the immune system to exhibit innate and adaptive responses to tumors and to maintain a memory of such responses, the “modern” immunotherapy with checkpoint inhibitors got remarkable results mainly in melanoma patients with the possibility to reach long-term benefit. This results were translated into different malignancies, and there are a lot of clinical studies still ongoing. However, important results include improvement in OS for NSCLC and RCC; these data have already well established in clinical practice. Moreover, promising results have been also obtained in head and neck, Hodgkin lymphoma, breast and ovarian cancer, neuroblastoma, and myeloma.

Data from ongoing clinical trials and further researches will provide more information about mechanisms of activation and resistance, as well as combination approaches. A critical issue will be also to provide biomarkers which could be predictive of response.

## Abbreviations

ADCC: Antibody-dependent cell- mediated cytotoxicity
AE: Adverse event
ASCT: Authologous stem cell transplantation
BRCA: Breast related cancer antigens
CCL: Chemokine ligand
CEA: Carcino-embryonic antigen
CSF-R1: Colony stimulating factor 1 receptor
CT: Cancer testis
CTL: Cytotoxic T lymphocyte
CTLA-4: Cytotoxic T lymphocyte antigen 4
DC: Dendritic cell
ELISA: Enzyme-linked immunosorbent assay
ELISPOT: Enzyme-linked immunospot
ER: Estrogen receptor
FDA: Food and Drug Administration
FOXO1: Forkhead box protein O1
GM-CSF: Granulocyte-macrophage colony-stimulating factor
HCC: Hepatocellular carcinoma
HER-2: Human epidermal growth factor receptor 2
HLA: Human leucocyte antigens
ICD: Immunogenic cell death
IFA: Incomplete Freund’s Adjuvant
IFNγ: Interferon gamma
irPFS: Immune-related progression-free survival
LAG-3: Lymphocyte-activation gene 3
MAGE-A3: Melanoma AntiGen Encoding-antigen 3
MDSC: Myeloid-derived suppressor cell
mTOR: Mammalian target of rapamycin
NK: Natural killer
NKT: Natural killer T cells
NSCLC: Non-small-cell lung cancer
OC: Ovarian cancer
ORR: Overall response rate
OS: Overall survival
PBMC: Peripheral blood mononuclear cell
PCR: Polymerase chain reaction
PD-1: Programmed cell death protein 1
PD-L1: Programmed death ligand 1
PFS: Progression-free survival
RCC: Renal cell cancer
RT: Radiotherapy
sdRNA: Seed region ribonucleic acid
TAA: Tumor-associated antigen
TGF: Transforming growth factor
TIL: Tumor-infiltrating lymphocyte
TLR: Toll-like receptor
TME: Tumor microenvironment
TNBC: Triple-negative breast cancer
T-reg: Regulatory T cells
T-VEC: Talimogene laherparepvec
VEGF: Vascular endothelial growth factor
